# Building effective service linkages in primary mental health care: a narrative review part 2

**DOI:** 10.1186/1472-6963-11-66

**Published:** 2011-03-25

**Authors:** Jeffrey D Fuller, David Perkins, Sharon Parker, Louise Holdsworth, Brian Kelly, Russell Roberts, Lee Martinez, Lyn Fragar

**Affiliations:** 1 School of Nursing and Midwifery, Flinders University, Adelaide, Australia; 2 Northern Rivers University Department of Rural Health, School of Public Health, University of Sydney, Lismore, Australia; 3 Broken Hill University Department of Rural Health, School of Public Health, University of Sydney, Broken Hill, Australia; 4 Research Consultant, Sydney, Australia; 5 School of Tourism & Hospitality Management, Centre for Gambling Education & Research, Southern Cross University, Lismore, Australia; 6 Faculty of Medicine, University of Newcastle, Newcastle, Australia; 7 Greater Western Area Health Service, Orange, New South Wales, Australia; 8 South Australian Department of Health, Adelaide, Australia; 9 Australian Centre for Agricultural Health and Safety, School of Public Health, Sydney University, Moree, Australia

**Keywords:** Narrative review, mental health services, primary health care, cooperative behaviour

## Abstract

**Background:**

Primary care services have not generally been effective in meeting mental health care needs. There is evidence that collaboration between primary care and specialist mental health services can improve clinical and organisational outcomes. It is not clear however what factors enable or hinder effective collaboration. The objective of this study was to examine the factors that enable effective collaboration between specialist mental health services and primary mental health care.

**Methods:**

A narrative and thematic review of English language papers published between 1998 and 2009. An expert reference group helped formulate strategies for policy makers. Studies of descriptive and qualitative design from Australia, New Zealand, UK, Europe, USA and Canada were included. Data were extracted on factors reported as enablers or barriers to development of service linkages. These were tabulated by theme at clinical and organisational levels and the inter-relationship between themes was explored.

**Results:**

A thematic analysis of 30 papers found the most frequently cited group of factors was "partnership formation", specifically role clarity between health care workers. Other factor groups supporting clinical partnership formation were staff support, clinician attributes, clinic physical features and evaluation and feedback. At the organisational level a supportive institutional environment of leadership and change management was important. The expert reference group then proposed strategies for collaboration that would be seen as important, acceptable and feasible. Because of the variability of study types we did not exclude on quality and findings are weighted by the number of studies. Variability in local service contexts limits the generalisation of findings.

**Conclusion:**

The findings provide a framework for health planners to develop effective service linkages in primary mental health care. Our expert reference group proposed five areas of strategy for policy makers that address organisational level support, joint clinical problem solving, local joint care guidelines, staff training and supervision and feedback.

## Background

The chronic and relapsing nature of many severe mental disorders, the complex needs of sufferers and their carers around stigma and isolation, and dissatisfaction with service access and quality have led governments to prioritise collaborative service delivery based in primary care [1-3]. Despite widespread availability, many primary care services have not worked collaboratively to meet mental health care needs [4]. While there is support for collaborative mental health services in primary care, it is not clear how these should be introduced, made to work effectively and sustained.

Factors conducive for collaboration include goal predictability (goals are known by all partners even when circumstances change), collective efficacy (agreement about goals and confidence in other partners) and role clarity [5,6]. These factors are hard to achieve when working with chronic health conditions across a range of helping services in community settings.

This narrative review was conducted to address national government priorities concerning improved service linkages in the Australian health care system. The first objective was to examine evidence from the international literature regarding the effectiveness of linkages in primary mental health care, which is reported in a part one companion to this paper. The second objective was to describe the factors that enable the development of these linkages, which is reported in this part two paper.

## Methods

The study followed the narrative review and thematic synthesis approaches designed to combine quantitative and qualitative evidence and support decision making by policy makers [7-9]. A reference group of eight senior policy and service managers in Australian primary mental health care was established to guide the review, help interpret the findings and assist in the formulation of recommendations. The method is fully described in the part one companion paper. The focus of this part two paper is on how to build effective service linkages. The conclusions draw on the interpretive insights of the reference group and the research team, as well as the international literature [8].

### Search strategy

A comprehensive search of biomedical, psychological and social databases was conducted of English language papers published between 1998 and March 2009 from Australia, New Zealand, Canada, USA and Europe. These databases included MEDLINE, Embase, Psychinfo, Cinahl, ProQuest, Sociological Abstracts, Family and Society Plus, Meditext and all Evidence Based Medicine (EBM) Reviews. Inclusion criteria were based on the following operational definitions of primary mental health care and primary mental health care linkages.

Primary Mental Health Care (PMHC) is:

1. Multi-faceted and comprising first level of contact, providing continuous care in a non-specialist setting. PMHC includes recovery, rehabilitation and ongoing support.

2. PMHC includes early intervention, treatment, health education and promotion for individuals as well as pathways to specialist care.

3. PMHC may include linkages with and referral between services in health (such as between a GP and mental health specialist) and non-health (such as a welfare service).

4. PMHC concerns clinical care to individuals involving a primary health care clinician. While PMHC can include population-wide health promotion, advocacy and community development, these were not included in this review.

A primary mental health care linkage was defined as follows:

1. The linkage is the process used to connect two or more services in the provision of clinical primary mental health care.

2. One part of the linkage must involve a primary health care practitioner such as a GP, community nurse or practice nurse. The other part of the linkage can be any health or human service entity including hospital or community based mental health specialists, private practitioners, or non-health agencies such as housing, education or welfare etc. Linkages must be two-way which excludes a single referral without feedback or continuing relationship.

### Inclusion criteria

Studies which reported on factors that enable or hinder linkages were included. These used a range of descriptive study designs including surveys and questionnaires and qualitative studies which captured the experience of participants in the linkage. The qualitative studies chiefly used interviews, but also focus groups, observation of meetings and analysis of referral letters. Some of the papers were part of the randomised trials reported in the part one paper and provided valuable contextual information from these studies.

The restriction of a linkage to a clinical service meant that the review focused on factors at the clinical level. Hence, wider policy and program supports, such as national infrastructure were not included and this is a limitation of our review.

### Data extraction & analysis

**Figure 1:**
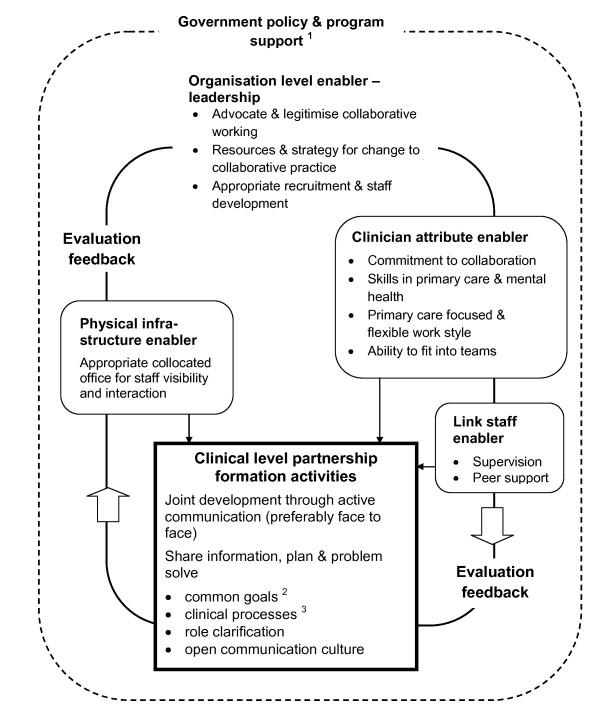
**Inter-relationship between developmental factors**. 1. Government policy & program support in shaded text to acknowledge this level but that is not included in this review 2. Involvement of patients in decision making enables patient-centred goals to be the common reference point for health care providers 3. Includes treatment plans, referral and follow up protocols

The data extraction template and coding framework are described in the part one companion paper. The papers coded as collaborative factors were further coded according to the enablers and barriers they described. Initial codes were agreed *a priori *based on the research team's knowledge and prior reading. The code list was further refined during data synthesis (JF & LH) to adjust for the use of different terms describing similar factors and to add new factors as required. Using this iterative process, the recurring themes on developmental factors in the studies were identified and refined through team and reference group discussions. The analysis comprised narrative synthesis whereby the factors were described and grouped by theme. This process involved tabulating the factors according to whether they were described as an enabler or a barrier to building service linkages (see additional file 1, appendix 1). We then theorised the inter-relationship between the factors through a graphical representation of the level at which they operate and whether they were a formation or a enabling activity (figure 1).

Since our review was designed to aid decision support for policy-making, the expertise of our policy maker and service director review group was particularly valuable as we considered recommendations for building service linkages. We first asked them to comment on how the findings from the international literature related to their experience in the Australian system and then, based on the factors identified, what they would recommend as acceptable, feasible and important actions to Australian health care policy makers.

## Results

From 158 citations in the review, 30 papers covering 26 studies addressed factors underpinning the development of service linkages. Our thematic analysis grouped findings at two levels: clinical and organisational. The clinical level concerns clinicians working together in planning, delivering or reviewing care to patients. The organisational level concerns the management of strategy, facilities and resources within and between collaborating services. Given the focus of our review on clinical linkages it is not surprising that most of the material concerned developmental factors at the clinical level (see additional file 1, appendix 1).

### Clinical level

At the clinical level we identified a set a factors describing activities that primary care and mental health clinicians would undertake together to form a partnership (linked service). We labelled these as "partnership formation activities" which we defined as the process of human organisation central to team building. We also identified further factors as support to partnership formation activities and we have mapped the inter-relationship of these factors in figure 1.

Three papers emphasised the need for all parties, mental health and primary care, to be equally involved in the development of the partnered service arrangements [10-12]. This was important for ensuring joint ownership and mutually beneficial outcomes. Nine papers described how active joint practitioner communication occurred, ideally in person and through regular clinical meetings, with information sharing and clinical problem solving, formulation of a common understanding about the nature of the partnership and its operation and a set of agreed goals. Active communication between practitioners included development of processes (e.g., care planning, guideline development, referral and follow up protocols) which enabled structured communication and communication channels such as regular meetings. This was helped by (and would help create) a receptive partnership culture, such as an "open door" communication style and a willingness to try out new ideas. A communication process also enabled a partnership to monitor and consider how it was operating to meet patients' needs [13-21].

In relation to goals, there was some evidence that patient involvement in care processes was also important. While only one paper specifically discussed the use of educational and behavioural change strategies to engage patients as collaborators with clinicians in setting their own goals and management of their care [18], many papers included these strategies in their intervention. Our reference group considered that patient set self-management goals could be a common focus for the different members of the clinical team. Hence the establishment of common goals as an aspect of clinical partnership formation may be enabled when the model of care includes patients as collaborators.

Joint development of a partnership through regular communication at the clinical level could assist the clarification of partner roles and attention to the different role concerns of the partners. Role clarity was the most frequently described aspect of partnership formation (14 papers), particularly as a barrier when this clarity was not present [16,18,19,22-32]. We have extracted data from a few of these studies to describe the types of role conflict and also how role clarification occurred. Different or conflicting expectations about roles were reported as a barrier by Yaffe [25] and Macdonald [24]. Yaffe et al surveyed patients, family doctors and psychiatrists in a Canadian psychogeriatric outpatient clinic over 18 months. They found that clinicians disagreed about responsibility for treatment in 40% of cases, with family physicians expecting the psychiatrist to provide care after referral, while psychiatrists considered referrals were for assessment only. Furthermore, half of the patients said they did not know what to expect from their consultation with the psychiatrist. Macdonald et al interviewed 75 primary mental health workers in the UK and found that some had been expected to design their own roles without guidance. These workers reported feeling like "the meat in the sandwich" between primary care staff, who wanted them to adopt a direct clinical role, and mental health staff, who wanted them to take a consultation liaison role.

Team role clarification processes in the US IMPACT trial enabled the identification of safe practice boundaries [18]. The activities that supported role clarification were weekly team meetings between the care manager (Depression Care Specialist), the primary care physician and the psychiatrist as well as regular peer support telephone conferences with other care managers to discuss their work. Frazer et al found similar benefits in a survey of 13 Primary Care Graduate Mental Health Workers in the UK, but also reported that the development of an Integrated Care Protocol was a key component of role clarification [22]. A Canadian evaluation of primary care counsellors found that regular meetings for mutual support were important, as was supervision of the counsellor by a mental health specialist (in this case a psychiatrist) for case-based discussion and making treatment recommendations [12]. Kirchner reported similar findings about supervision in the US PRISM-E trial [17].

Another barrier to role clarity between primary care and mental health services concerned referrals, where there were different expectations about role responsibility for patients and dissatisfaction with referral processes [16,27-32]. When primary care physicians referred large numbers of patients with "low severity" conditions to mental health teams, then this created an excessive demand on the team [28]. The team labelled many of these referrals as "inappropriate", often without considering the physician's needs or referral threshold [29,30]. Chew-Graham and Slade reported on the failure of a validated Threshold Assessment Grid designed to aid referrals, as it was used by only 25% of physicians and few mental health teams [30,32]. The clinicians found the Grid too simplistic and reductive. This was in a context without clear referral processes and tension about whether the mental health team role was crisis-response or caring for those with long term mental illness. Primary care physicians wanted direct access to psychiatrists and they felt that mental health team triage and the use of the Grid hindered access and undermined the doctor-to-doctor relationship [30,31].

In addition to the supervision and mutual support of staff involved in linkage roles mentioned above, other factors were also described as supporting partnership formation. Five papers mentioned that an ideal attitude and skill set for staff was knowledge and skills in both mental health and primary care [14,17,18], a flexible work style that helped a person fit into the team [12,17,19] and a belief that collaboration is worthwhile [14]. However, one study found that difficulty in recruiting experienced mental health workers who were willing to take on collaboration roles was a barrier to collaborative care [33].

The physical features of a clinic were reported as an enabler, when the office of the care manager (Depression Care Specialist) was located in the primary care clinic for optimum team visibility and interaction [18], but a barrier when room space for the care manager (Primary Mental Health Link Worker) was inadequate [24]. Primary care physicians in larger practices of at least four physicians were found to be more amenable to collaborative care, made more referrals to community mental health teams and fewer referrals to in-patient and out-patient psychiatric services. These differences were attributed to consultation liaison arrangements and co-located psychology services in these larger clinics [26,34]. Another study found, however, that the number of primary care physicians working in a clinic made no difference to their satisfaction in communication with psychiatrists [35].

A feature of papers that reported active joint practitioner communication processes was the inclusion of evaluation feedback in these processes. One of the largest trials in our review came to an explicit conclusion that mechanisms to feed back evidence about outcomes to partners was "*the most important factor [for sustainability], cited in four of the five IMPACT study sites*". These mechanisms included a reliable patient tracking, information and communication process to the team [26].

### Organisational level

At the organisational level, five papers described the legitimisation of a collaborative approach through a supportive institutional environment of leadership and a change management for linked mental health services [17,26,33,36,37]. In the IMPACT study by Blasinsky et al, key informants indicated that strategic organisational leadership for collaborative care was important for the sustainability of the collaborative service model [26]. Our reference group also considered that the authority embodied in leadership can promote the legitimacy of collaborative practice. This may be important to counter resistance, such as that reported by Richards et al who found some concern from GPs that collaborative practice could challenge their leadership of patient primary care [33].

In the PRISM-E study, Kirchner et al compared leadership in two integrated care clinics, one that had successfully achieved integration and the other that had not [17]. The "unsuccessful" clinic had undergone change, which was perceived as chaotic and overwhelming. Part of this change included the employment of a new and influential leader who did not support integration, which subsequently led to "turf disputes" amongst the clinic services.

While we have proposed that partnership formation at the clinical level is necessary for the development of linked clinical services, this is unlikely to be sufficient to address organisation wide barriers, such as narrow and different service mandates and priorities that fail to address continuity of care, un-pooled resources and poor connections between senior managers across services. Discussing these wider organisational barriers, Rees et al concluded that organisational leadership is important to ensure accountability mechanisms are developed, to influence strategy and to provide resources for the change to collaborative practice [37].

## Discussion

In this section we first discuss the inter-relationship between developmental factors described above (see figure 1) and then five recommended areas of strategy for policy makers. The core factors are clinical level partnership activities that clinicians undertake to develop linked service delivery. These partnership formation activities are ideally conducted in an organisational context with strong leadership support. Three other set of factors were found to enable partnership formation: having staff with the right attributes for collaboration in primary mental health care; the provision of supervision and peer support to staff involved in making linkages; and provision of office accommodation conducive to collaboration. Influencing all partnership formation activities and supports is an evaluation feedback loop that serves as a motivation enabler for sustaining collaboration.

From our analysis of these studies and our reference group discussions on policy importance, acceptability and feasibility, we identified five strategies for policy makers and service directors to promote partnership formation and hence build service linkages in primary mental health care:

1. Provide organisational level support for integration.

2. Facilitate joint clinical planning and problem solving.

3. Jointly develop local care guidelines (crisis plans, referral protocols and follow up arrangements) through regular meetings and the use of a common planning process.

4. Provide training, support and supervision of staff committed to work in primary care and mental health.

5. Feedback evidence about outcomes to service partners.

Our conclusion about organisational level support for integration comes from the study findings, but also from the observation that the research trials reported systematic interventions, with specific funding, leadership and change management support. Such organisational level supports are also required if the findings of this review are to be implemented successfully. This is supported by the intervention research literature, such as by Damschroder et al, who concluded that translation of effective models of care from research into everyday practice requires consideration of the intervention characteristics; the economic, social and political context; the structural, political and cultural contexts of the organisations involved; the agency and skills of the individual involved; and the implementation change management process at clinical and organisational levels [38]. Hence, to address even some of these domains, specific organisational level supports are needed in non study settings.

Since greater role ambiguity is inherent in collaborative work, particularly in mental illness where a patient's condition can change between acute, chronic and recovery phases, attention is required to problem solving and the clarification of roles. Our findings indicate that a process to form linkages and deal with role issues, which is generalisable in different contexts, is joint clinical planning and problem solving. This is a large component of partnership formation activities as described in the literature. This planning and problem solving can occur between clinicians when they discuss patient care and as they consider linked service models, thereby developing ongoing personal links and professional relationships and building mutual trust. The immediate gains from such clinically grounded discussions could be the reinforcement needed to motivate staff to make a sustained effort to collaborate. The Australian Fourth National Mental Health Plan notes that dealing with role tension about activities, such as transporting patients with mental illness, access to in-patient care and management of people who are intoxicated, can be jointly resolved in this way [3]:

*How such tensions are resolved will depend on the development of local solutions backed by good collaboration between sectors and recognition of roles, responsibilities and limitations. Patients and carers should routinely be involved in such deliberations*. (2009:42)

Joint clinical planning and problem solving may help to form professional relationships between mental health and primary care services. This may counter service stigma and resistance that is evident in the finding that some GPs have limited interest in providing mental health care, feel under trained and do not see this as their role [39]. In order to promote joint clinical problem solving between primary care and mental health their leaders will require skills, a clear remit and the necessary resources.

We also found that joint clinical problem solving should work on agreed service arrangements. Joint development of linked services arrangements is important to ensure that the model of care meets the needs of primary care and mental health providers, as well as patients and carers. Since contexts differ, local joint planning and problem solving must be flexible to account for these differences. Planning could cover such processes as documented referral processes (communication); care policies and procedures (guidelines); mechanisms for regular team leader and service-wide meetings; strategies for inclusion of patient and carer needs in decision making; and care coordination to ensure that the patients and services are linked. The finding that larger primary care practices were more amenable to collaborative care is relevant, since more staff requires that more attention be given to the development of agreed service arrangements. As a consequence larger primary care practices may have been able to negotiate referral and shared care arrangements with mental health services. Hence, local joint planning and problem solving is more likely to be relevant and sustainable if pitched at practices or collection of practices with four or more primary care physicians.

Our findings suggest that flexibility, commitment and skills to work in mental health and primary care, and motivation towards teamwork are staff attributes that help develop competence in collaboration. This is particularly the case when the workplace is receptive to change [14,26], when staff have appropriate supervision from an experienced mental health clinician and peer support [24,26,40] and when their work is supported by a care plan or guidelines [8-10]. However, the ability to work in a collaborative problem-solving manner between primary care and mental health is a personal competency that has not been included in the formal training of primary care providers or mental health staff. While this competency might be achieved through "on-the-job" problem solving noted above, this would also be helped through inter-professional training at undergraduate and postgraduate levels. Our findings also support the value of expert supervision or mentorship [26], as well as forums for mutual support for those staff undertaking linkage roles [24]. These strategies could increase the mental health skills of these staff under expert guidance, as well as develop their role with others as they jointly identify role issues that require clarification.

Given the finding that feedback of evidence about service outcomes is an important driver of change, then the development of mental health data collection and systems for accountability are timely. Accountability can be articulated at the level of policy (for government and management) and clinical care (for service providers). Outcome data could be considered against national standards for mental health and primary care services, but these data must cover the key links between mental health services, primary care services and the wider human service sectors [41,42]. The collection and reporting of data requires resources, has opportunity costs and may require new information and communication mechanisms. However, if communities are to have evidence-based and responsive primary mental health care services then such accountability must be resourced. Collection of such data across sectors would be aided by common patient identifiers, electronic health records and patient enrolment with a primary care provider [43].

### Limitations

We did not conduct a quality assessment of the retrieved studies as they reported methods and results in different ways, with different theoretical perspectives and varying levels of detail. This range of studies in our broad review (over the two parts) would have made applying comparative quality criteria complex and outside our funded timeframe of one year. This is a limitation of our findings, as we cannot give weight to these findings beyond an estimate of importance of each factor based on the number of studies in which they were reported.

While the findings from this review are informative, about the factors that enable the formation of mental health service partnerships in primary care, the multifactoral and context dependent nature of these factors does limit the generalisation of the findings. This would particularly be the case when considering whether findings from one country apply to another.

## Conclusion

This review has identified key factors that health planners should consider if they want to develop service linkages in primary mental health care. These factors include: joint clinical team problem solving; staff attributes, capacity and support; physical infrastructure; planning and decision making based on evidence of outcomes; and supportive leadership. Based on these findings we summarise what can be done to build service linkages in primary mental health care under three broad recommendations. The first is to build collaborative mental health planning and problem solving mechanisms, particularly at the clinical service delivery level, supported by local and regional management levels. The second is to increase workforce capacity related to the attitudes, skills and confidence in working in partnership models in order to meet competency standards in collaborative mental health care, The third is to develop performance indicators for collaboration and then to collect and publish data that describes the performance of integrated primary mental health services against these indicators. Such reporting would provide the motivation to maintain successful collaboration.

There is no question that research is needed to improve service linkages between primary and secondary providers of mental health services using a comprehensive model of implementation. To date this has been a secondary consideration and so the translation of services models from research to practice has been largely absent and which our review has sought to address.

## Competing interests

DP was an author of one included study but there were no other conflicts of interest. The authors declare that they have no other competing interests.

## Authors' contributions

JF & DP jointly coordinated the review, conducted the data extraction and analysis and participated in the drafting of this paper. SP & LH conducted the search, data extraction, analysis and participated in the drafting of this paper. BK, LF, LM & RR advised on the conduct of the review and analysis and participated in the drafting of this paper. All authors read and approved the final manuscript.

## Pre-publication history

The pre-publication history for this paper can be accessed here:

http://www.biomedcentral.com/1472-6963/11/66/prepub
